# Long-Term Effects of Repetitive Mild Traumatic Injury on the Visual System in Wild-Type and TDP-43 Transgenic Mice

**DOI:** 10.3390/ijms22126584

**Published:** 2021-06-19

**Authors:** Kristina Pilipović, Jelena Rajič Bumber, Petra Dolenec, Nika Gržeta, Tamara Janković, Jasna Križ, Gordana Župan

**Affiliations:** 1 Department of Basic and Clinical Pharmacology and Toxicology, Faculty of Medicine, University of Rijeka, Braće Branchetta 20, 51 000 Rijeka, Croatia; kristina.pilipovic@medri.uniri.hr (K.P.); jelena.rajic@medri.uniri.hr (J.R.B.); petra.dolenec@medri.uniri.hr (P.D.); nika.grzeta@medri.uniri.hr (N.G.); tamara.jankovic@medri.uniri.hr (T.J.); 2 Department of Psychiatry and Neuroscience, Faculty of Medicine, University Laval, Québec City, QC G1V 0A6, Canada; jasna.kriz@fmed.ulaval.ca

**Keywords:** brain injuries, traumatic, diffuse axonal injury, geniculate bodies, mice, nerve degeneration, neurofilament proteins, neuroglia, optic tract, superior colliculi, synaptophysin, TDP-43 proteinopathies

## Abstract

Little is known about the impairments and pathological changes in the visual system in mild brain trauma, especially repetitive mild traumatic brain injury (mTBI). The goal of this study was to examine and compare the effects of repeated head impacts on the neurodegeneration, axonal integrity, and glial activity in the optic tract (OT), as well as on neuronal preservation, glial responses, and synaptic organization in the lateral geniculate nucleus (LGN) and superior colliculus (SC), in wild-type mice and transgenic animals with overexpression of human TDP-43 mutant protein (TDP-43^G348C^) at 6 months after repeated closed head traumas. Animals were also assessed in the Barnes maze (BM) task. Neurodegeneration, axonal injury, and gliosis were detected in the OT of the injured animals of both genotypes. In the traumatized mice, myelination of surviving axons was mostly preserved, and the expression of neurofilament light chain was unaffected. Repetitive mTBI did not induce changes in the LGN and the SC, nor did it affect the performance of the BM task in the traumatized wild-type and TDP-43 transgenic mice. Differences in neuropathological and behavioral assessments between the injured wild-type and TDP-43^G348C^ mice were not revealed. Results of the current study suggest that repetitive mTBI was associated with chronic damage and inflammation in the OT in wild-type and TDP-43^G348C^ mice, which were not accompanied with behavioral problems and were not affected by the TDP-43 genotype, while the LGN and the SC remained preserved in the used experimental conditions.

## 1. Introduction

Repetitive mild traumatic brain injury (mTBI) represents a current and growing serious medical and economic problem worldwide. It is particularly common in athletes engaged in contact sports, such as soccer, ice hockey, American football, boxing, wrestling, and mixed martial arts [1,2], as well as in victims of domestic spousal violence or child abuse [3] and military personnel [4,5]. The true prevalence of repetitive mTBI is not known because the symptoms of a single mTBI or concussion frequently resolve without medical care, pass spontaneously, and stay unrecognized, unreported, or undiagnosed. For example, in most patients, especially adult athletes, some of the post-concussion symptoms, such as dizziness, disorientation, confusion, or headache, subside within 10 days [6] and, in some cases, within several months following the first head trauma without specific interventions [7]. Increasing evidence suggests that, in humans or experimental animals with prior mTBI history, the susceptibility to brain damage induced by a future TBI is augmented and that repetitive injuries have cumulative effects, enhancing a risk for long-term and later-life cognitive, behavioral, and psychiatric disturbances, as well as the development of neurodegeneration [8,9,10]. 

Repetitive mTBI has long been recognized as a risk factor for chronic traumatic encephalopathy (CTE), a condition characterized by generalized cerebral atrophy associated with widespread deposits of phosphorylated tau protein occurring as neurofibrillary tangles, diffuse beta-amyloid deposits, neuroinflammation, axonal pathology through the brain, and, in the majority of cases, by transactivation response element (TAR) DNA/RNA-binding protein 43 (TDP-43) immunoreactive intraneuronal and intraglial inclusions [10,11,12,13].

TDP-43 is predominantly a nuclear protein with the primary amino-acid structure similar to the members of the heterogeneous ribonucleoprotein family that shuttles between nucleus and cytoplasm [14]. Its intracellular functions in physiological conditions are insufficiently characterized, but it is becoming increasingly evident that TDP-43 is involved in specific pre-mRNA splicing and transcription events, in the regulation of mRNA stability, transport, translation, and degradation, and in chromatin condensation [14,15,16].

Recent evidence suggests that TDP-43 proteinopathy has been identified not only in CTE, but also in most cases of amyotrophic lateral sclerosis (ALS) [17], in a subset of the frontotemporal lobar degeneration (FTLD) with tau-negative ubiquitin-positive TDP-43-positive inclusions [18,19], and in specific disorders such as Alzheimer’s disease [20], Lewy body disease [21], hippocampal sclerosis [22], and corticobasal degeneration [20], suggesting its important role in the pathogenesis of neurodegeneration [23].

While TDP-43 dysregulation and accompanying neuropathological changes were documented in humans with previous history of repetitive mTBI [11], to our knowledge, they were investigated in only four published experimental studies. Elevated TDP-43 expression levels in the whole-cell lysates from the injured mouse cortical and hippocampal tissue [24,25], as well as the protein changes in the rat brain following blast TBI [26], were described. We detected transitory TDP-43 cytoplasmatic translocation and overexpression of the protein and its pathological forms in the frontal cortex within the first week following repetitive mTBI in mice [27]. Neurodegeneration and gliosis in the optic tracts (OT) of injured wild-type mice and animals with overexpression of human mutant TDP-43 protein (TDP-43^G348C^), a model of ALS/FTLD [28], were also demonstrated [27]. In addition, the level of damage in the OT was significantly increased in TDP-43 transgenic animals compared with wild-type mice at the end of the first week after the last injury [27]. TDP-43^G348C^ mice used in the mentioned study were 9–11 week old animals at the beginning of the study and did not show any neurodegenerative and behavioral impairments before head traumas. Here, we expanded our previous research to investigate the changes in the OT, as well as in the lateral geniculate nucleus (LGN) of the thalamus and the superior colliculus (SC), the brain structures that receive input from axons traveling in the OT, in wild-type and TDP-43^G348C^ animals at 6 months after the last brain trauma. We were interested in the level of neurodegeneration and glial activity in the OT and the mentioned nuclei, the presence of axonal injury and demyelination in the OT, and the possible synaptic changes included in visual information processing from retinal ganglion cells toward the nuclei. Chronic pathological changes in the OT, the LGN, and the SC of transgenic TDP-43 animals have not yet been studied. Furthermore, to the best of our knowledge, the preservation, glial responses, and synaptic organization in the mentioned nuclei of the visual pathway in wild-type mice in a model characterized with unconstrained head and body movements following the brain traumas have not been previously investigated. Moreover, because of different visual impairments detected in patients after mTBI [29], spatial learning and memory testing, which require preserved visual information processing, was conducted in mice of both genotypes. Such behavior in TDP-43 transgenic mice after repetitive mTBI has not been previously examined. Therefore, considering the results of our previous study and the fact that behavioral and the pathological brain changes are present for months after the initial injuries [12,27,30,31,32,33], this research hypothesized that the damage, gliosis, and synaptic reorganization in the OT and the investigated nuclei, as well as behavioral impairments induced by repeated mTBI, would be detected 6 months after the last injury and that they would be more pronounced in TDP-43^G348C^ mice. 

## 2. Results

### 2.1. Repetitive mTBI Induced Neurodegeneration, Axonal Injury, and Gliosis in the Optic Tract in Wild-Type and TDP-43^G348C^ Mice at 6 Months Following the Last Head Impact

In the first part of the study, we investigated whether repetitive mTBI causes the OT pathology 6 months after the last hit in wild-type and TDP-43 transgenic mice. We were especially focused on neuronal and axonal degeneration, demyelination, and glial activity in the mentioned brain structure after repeated brain impacts in the animals of both genotypes.

**Figure 1:**
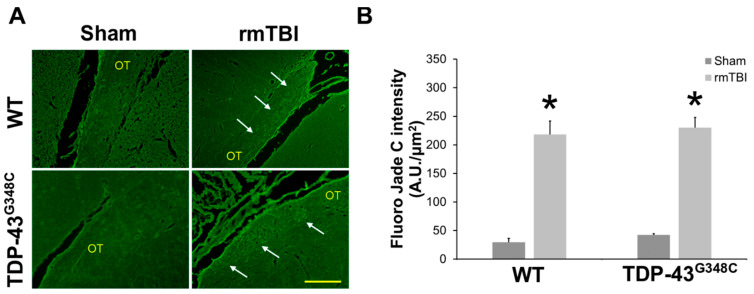
Neurodegeneration in the optic tract (OT) of wild-type (WT) and TDP-43^G348C^ mice at 6 months after repetitive mild traumatic brain injury (rmTBI). (**A**) Representative microphotographs of the OT stained with the Fluoro-Jade C fluorescent dye. Arrows point to Fluoro-Jade C-positive staining. Scale bar: 100 μm. (**B**) The histogram shows the intensity levels of the fluorescent staining (AU/μm^2^) in the OT of WT and TDP-43^G348C^ mice of the control groups (Sham) and animals with rmTBI. Results are expressed as means ± SEM (*N* = 5). * *p* < 0.05, significantly different from the related Sham.

Fluoro-Jade C was used as the marker of neurodegeneration [34,35]. This dye was found to stain degenerating nerve cell bodies and distal dendrites, axons, and terminals [36]. Figure 1A shows representative microphotographs of Fluoro-Jade C-stained sections of the OT in the sham animals and the traumatized mice of both genotypes. It is evident that Fluoro-Jade C-positive staining was detectable in the OT of the traumatized mice, both wild-type and TDP-43 transgenic, but not in the sham animals. Quantitative analysis of Fluoro-Jade C staining intensity confirmed these observations (Figure 1B). It was demonstrated that Fluoro-Jade C intensity levels were significantly higher in traumatized wild-type and TDP-43^G348C^ animals than in the sham animals of the corresponding control groups (*p* = 0.012; *p* = 0.012). No significant differences were observed between the levels of Fluoro-Jade C-positive staining in the OT of wild-type and transgenic TDP-43 injured mice (*p* = 0.676) (Figure 1B). 

**Figure 2:**
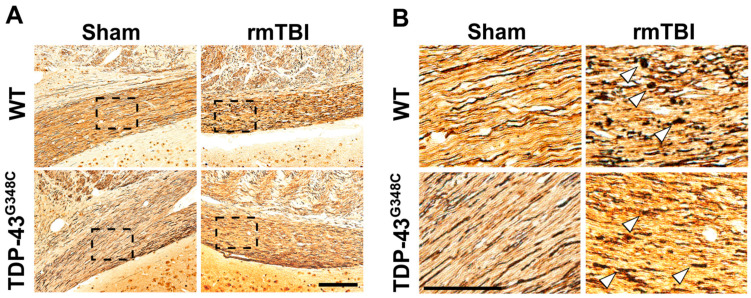
Axonal degeneration in the optic tract (OT) of wild-type (WT) and TDP-43^G348C^ mice at 6 months after repetitive mild traumatic brain injury (rmTBI). (**A**) Representative microphotographs of the OT stained with the silver staining in the mice of the control group (Sham) and the animals with rmTBI. Scale bar: 200 μm. (**B**) Microphotographs are higher-magnification images of the areas in the boxes of the corresponding panels. Arrowheads point to the spheroids of degenerating axons. Scale bar: 50 μm.

Silver staining is a method that has been used for visualization and localization of degenerating axons [37]. Representative microphotographs of silver-stained sections of the OT in mice of all experimental groups are shown in Figure 2. There was an increased silver uptake and staining in the OT of traumatized mice of both genotypes demonstrating evidence of axonal abnormalities compared with the sham-treated animals (Figure 2A). Furthermore, spheroids, a sign of axonal swelling, were observed in the axons of the OT in the injured wild-type and TDP-43 transgenic mice (Figure 2B). Results shown in Figure 2 suggest axonal injury in the OT at 6 months following repetitive mTBI in mice of both genotypes.

In order to analyze the integrity of myelinated neuronal fibers in the OT, we used conventional histological methods, i.e., staining with luxol fast blue (LFB) and immunohistochemical staining with anti-myelin basic protein (MBP) antibody.

**Figure 3:**
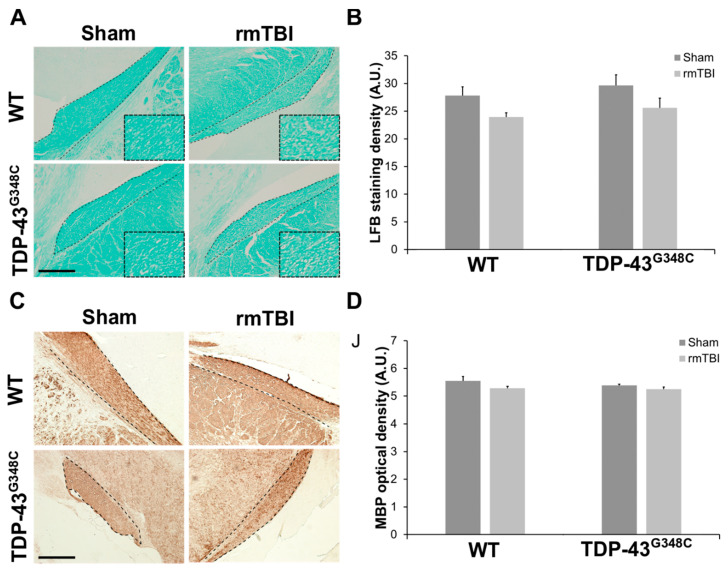
The integrity of myelinated neuronal fibers in the optic tract (OT) of wild-type (WT) and TDP-43^G348C^ mice at 6 months after repetitive mild traumatic brain injury (rmTBI). (**A**) Representative microphotographs of the OT stained with luxol fast blue (LFB). Higher magnification of the boxed regions reveals an area with reduced myelin and characterized by porous and weaker LFB staining. Dashed lines indicate the OT. Scale bar: 200 μm. (**B**) The histogram shows the LFB staining density (AU) in WT and TDP-43^G348C^ mice with rmTBI and related control groups (Sham). Results are expressed as means ± SEM (*N* = 4–6). (**C**) Representative microphotographs of the OT sections immunostained with anti-myelin basic protein (MBP). Dashed lines indicate the OT. Scale bar: 200 μm. (**D**) The histogram shows the MBP optical density (AU) in WT and TDP-43^G348C^ mice with rmTBI and related control groups (Sham). Results are expressed as means ± SEM (*N* = 4–5).

Representative photomicrographs of the OT sections stained with LFB in the traumatized wild-type and TDP-43^G348C^ mice, as well as in the sham animals, are shown in Figure 3A. It is evident that myelin density and integrity of myelinated fibers were approximately equal in sham-treated wild-type and transgenic TDP-43 animals, while reduced myelin in some parts of the OT, characterized by porous and weaker LFB staining, was detectable in traumatized mice of both genotypes (Figure 3A). Quantitative analysis demonstrated that the staining densities in the OT of the injured wild-type and TDP-43^G348C^ mice were slightly decreased in comparison to the levels in the related sham animals, but a statistically significant difference was not detected (*p* = 0.097). In addition, a significant difference in the levels of the LFB staining between the traumatized wild-type and TDP-43^G348C^ transgenic mice was also not revealed (Figure 3B).

Representative photomicrographs of the coronal OT sections that were stained with anti-MBP antibody and the quantitative analysis of the MBP optical density for the mice of all experimental groups are shown in Figure 3C,D. There were no differences in the MBP immunoreactivity (Figure 3C) and optical density (Figure 3D) between the traumatized groups and their related control groups for both genotypes or between the injured wild-type and TDP-43 transgenic animals (*p* = 0.234) at 6 months after the last head trauma. The results obtained by LFB and MBP staining suggest that the myelination of surviving axons in the OT was mostly preserved at the investigated time point after the last brain trauma.

**Figure 4:**
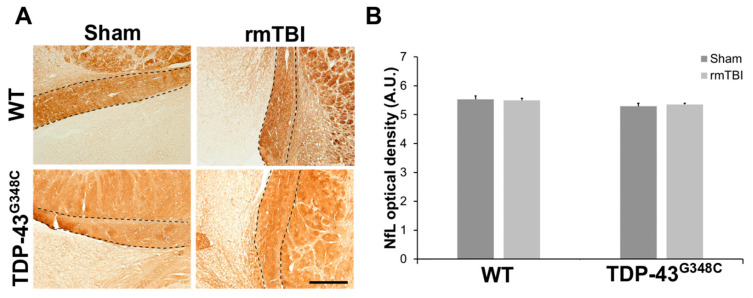
Neurofilament light chain (NfL) expression in the optic tract (OT) of wild-type (WT) and TDP-43^G348C^ mice at 6 months after repetitive mild traumatic brain injury (rmTBI). (**A**) Representative microphotographs of the OT stained with anti-neurofilament light chain protein. Dashed lines indicate the OT. Scale bar: 200 μm (**B**) The histogram shows NfL optical density (AU) in the axons of the OT in WT and TDP-43^G348C^ mice with rmTBI and related control groups (Sham). Results are expressed as means ± SEM (*N* = 3–5).

The integrity of surviving axons of retinal ganglion cells following repetitive mTBI was also examined using immunohistochemistry for neurofilament light (NfL) chain that is a major constituent of the neuronal cytoskeleton. Approximately equal NfL-positive staining was revealed in the axons of the OT in the traumatized and the sham mice of both genotypes (Figure 4A), while a significant difference in the NfL optical densities between the experimental groups was not revealed (*p* = 0.254) (Figure 4B). These results suggest that the NfL expression in the axons of the OT was unchanged at 6 months following repetitive mTBI.

**Figure 5:**
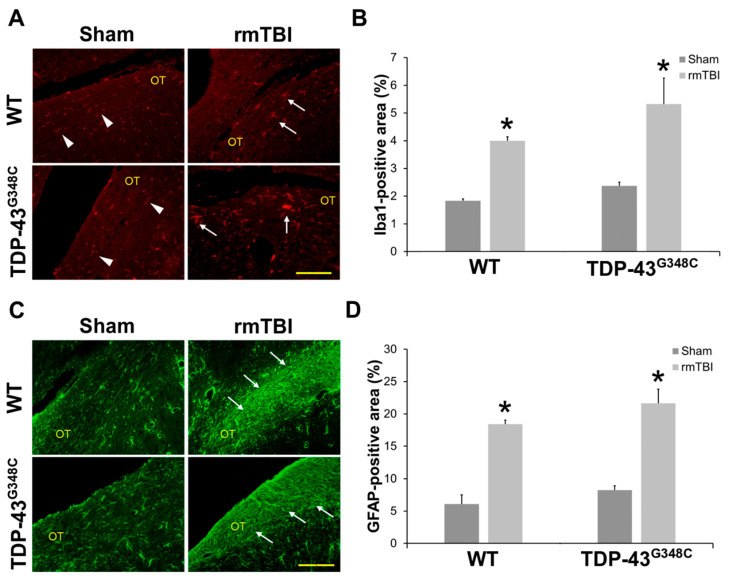
Gliosis at 6 months after repetitive mild traumatic brain injury (rmTBI) in the optic tract (OT) of wild-type (WT) and TDP-43^G348C^ mice. (**A**) Representative microphotographs of the OT immunostained with anti-ionized calcium-binding adaptor molecule 1 (Iba1). Arrowheads point to Iba1-positive cells with “resting” microglial morphology, and arrows point to Iba1-positive cells with activated microglial morphology. Scale bar: 100 μm. (**B**) The histogram shows the Iba1-positive area (%) in the OT of WT and TDP-43^G348C^ mice with rmTBI and related control groups (Sham). Results are expressed as means ± SEM (*N* = 5). * *p* < 0.05, significantly different from the related Sham. (**C**) Representative microphotographs of the OT immunostained with anti-glial fibrillary acidic protein (GFAP). Arrows point to the GFAP-positive cells with hypertrophic morphology. Scale bar: 100 μm. (**D**) The histogram shows the GFAP-positive area (%) in the OT of WT and TDP-43^G348C^ mice with rmTBI and related control groups (Sham). Results are expressed as means ± SEM (*N* = 5). * *p* < 0.05, significantly different from the related Sham.

The activity of the glial cells in the OT was examined using microglial marker ionized calcium-binding adaptor molecule 1 (Iba1) and the astrocytic marker glial fibrillary acidic protein (GFAP) (Figure 5).

Figure 5A shows representative microphotographs of the Iba1 immunostained OT sections in mice of all the experimental groups. “Resting” microglia, i.e., the microglial cells with thin Iba1-immunoreactive processes, were detected in the sham-treated mice of both control groups. Moreover, in all the traumatized animals, activated microglia, i.e., microglial cells with hypertrophic and large cell bodies, thick processes, and with amoeboid and migrating morphology, were noticed (Figure 5A). The higher magnifications images of “resting” and “activated” microglia are shown in Figure S1 (Supplementary Materials).

Quantitative analysis of the percentages of the Iba1 immunoreactive areas in the OT demonstrated statistically significant higher values in the injured wild-type and TDP-43^G348C^ animals compared with related sham mice (*p* = 0.012; *p* = 0.012) (Figure 5B). However, a statistically significant difference in the percentages of the Iba1 immunoreactive areas between the traumatized wild-type and TDP-43 transgenic mice was not found (*p* = 0.296) (Figure 5B).

Morphological changes that would suggest astroglial activation were not detected in the OT of the sham injured wild-type and TDP-43^G348C^ mice at 6 months after the last head trauma (Figure 5C). Contrarily, the GFAP immunoreactivities were more pronounced in the OT of traumatized wild-type and TDP-43 transgenic mice than in sham-treated animals, suggesting astrocytic hypertrophy after repetitive mTBI (Figure 5C). Furthermore, the percentages of the OT areas covered by hypertrophic astrocytes in the traumatized animals of both genotypes were significantly higher compared with the values noticed in the non-injured mice (*p* = 0.012; *p* = 0.012) (Figure 5D). In addition, the values of GFAP-positive areas in the injured TDP-43^G348C^ animals did not significantly differ from the values in wild-type mice (*p* = 0.210) (Figure 5D).

### 2.2. Repetitive mTBI Did Not Cause Neurodegeneration, Glial Activation, and Synaptic Reorganization in the Lateral Geniculate Nucleus and the Superior Colliculus in Wild-Type and TDP-43^G348C^ Mice at 6 Months Following the Last Head Impact

The presence of neurodegenerative changes in the LGN and the SC, the regions that receive direct innervation from the retinal ganglion cells via the OT, were analyzed using Fluoro-Jade C and cresyl-violet staining. From the representative microphotographs of Fluoro-Jade C-stained sections of the LGN, shown in Figure S2A (Supplementary Materials), it is evident that the staining used was not detected in any of the experimental animals, suggesting no neurodegeneration in this structure at 6 months after the last mTBI. Moreover, individual Fluoro-Jade C-positive staining was detectable in the superficial SC of the injured mice of both genotypes (Figure S2D, Supplementary Materials). Quantitative analysis demonstrated a slight increase in the Fluoro-Jade C intensity levels in the SC of the traumatized wild-type and TDP-43^G348C^ animals compared with sham control animals. However, a statistically significant difference between the experimental groups was not revealed (*p* = 0.235) (Figure S2E, Supplementary Materials).

Additionally, cresyl-violet staining revealed approximately equal neuronal cell density in the LGN (Figure S2B, Supplementary Materials) and the SC (Figure S2F, Supplementary Materials) of all the experimental groups of mice, which was confirmed by subsequent quantitative analysis (*p* = 0.113, *p* = 0.617) (Figure S2C,G, Supplementary Materials).

Repetitive mTBI did not cause changes in the activity of the glial cells in the LGN and the SC of wild-type and TDP-43 transgenic mice in our experimental conditions (Figure S3, Supplementary Materials). Specifically, in the investigated nuclei of the traumatized and sham-treated mice of both genotypes, the “resting” but not activated microglia was detected (Figure S3A,D, Supplementary Materials). Furthermore, a statistically significant difference in the number of Iba1-positive cells between the groups was not revealed for the LGN (*p* = 0.200) or for the SC (*p* = 0.446). Moreover, the signs of astrocytosis in the examined nuclei were not detected in any of the experimental animals (Figure S3C,F, Supplementary Materials).

To detect if repetitive mTBI affects synaptic density in wild-type and TDP-43^G348C^ animals, anti-synaptophysin (SYP) immunostaining was performed. Although it seemed to be more pronounced in the LGN of the sham and injured TDP-43^G348C^ animals compared with wild-type mice (Figure S4A, Supplementary Materials), a significant difference between the experimental groups in the SYP staining intensities was not obtained (*p* = 0.069) (Figure S4B, Supplementary Materials). Moreover, significant differences in the SYP expression (Figure S4C, Supplementary Materials) and density intensities (Figure S4D, Supplementary Materials) between the groups were not observed in the SC (*p* = 0.100).

### 2.3. Repetitive mTBI Did Not Affect the Barnes Maze Task in Wild-Type and TDP-43^G348C^ Mice at 6 Months Following Repetitive mTBI

**Figure 6:**
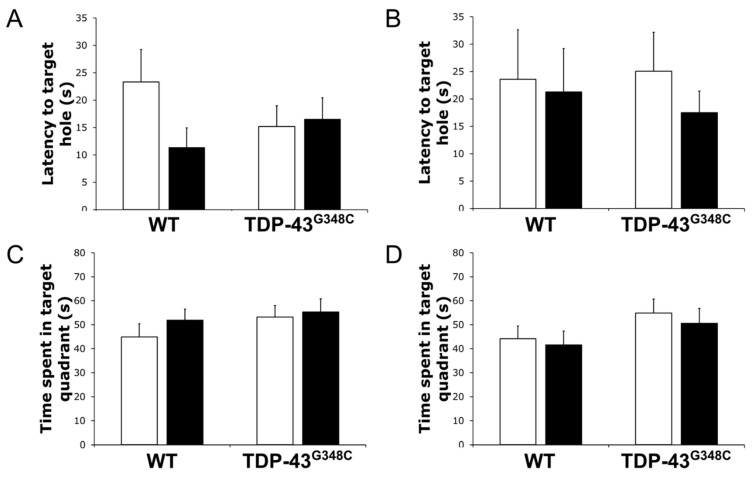
Barnes maze task performance in wild-type (WT) and TDP-43^G348C^ mice at 6 months after repetitive mild traumatic brain injury (rmTBI). The histogram shows the latency time (s) to reach the target hole during (**A**) the tests and (**B**) the retests or the time (s) spent in the target quadrant during (**C**) the tests and (**D**) the retests for mice with rmTBI (■) and related control groups (□). Results are expressed as means ± SEM (*N* = 12–14).

To explore the long-term effects of repetitive mTBI on the function of the visual system, we trained experimental animals in the Barnes maze. Figure 6 shows the latency time to reach the target hole and the time spent inside the target quadrant during the tests and the retests for all the experimental groups of mice. There was no significant difference in the latency time to reach the target hole between the experimental groups of mice during the tests (*p* = 0.148) and the retests (*p* = 0.763) (Figure 6A,B). Additionally, no statistically significant differences in the time spent in the target quadrant between the experimental animals were obtained for the tests (*p* = 0.527) and the retests (*p* = 0.381) (Figure 6C,D).

## 3. Discussion

Different visual problems, such as blurred vision, visual acuity loss, and visual field defects, have been reported in patients with previous history of moderate to severe TBI [38], but much less is known about these impairments and pathological changes in the visual system following mild and especially repetitive mTBI [39]. Studies exploring neuropathological changes in the visual system in animal models of repeated head traumas are scant [27,40,41,42,43]. The aims of this study were focused on the chronic effects of repetitive mTBI on some parts of the visual system in wild-type and TDP-43^G348C^ mice. As in our previous study [27], we used the abovementioned mice with overexpression of human familial ALS-linked mutant TDP-43 protein and a predisposition to the pathological accumulation of its aggregates, cytotoxic cleavage fragments, axonopathy, and neuroinflammation in the brain and the spinal cord [28]. The mentioned pathological and biochemical changes are age-related [28]. Thus, the TDP-43 ^G348C^ mice did not develop TDP-43-positive aggregates at an early age of 9–11 weeks, as they were at the beginning of the study. The TDP-43-positive aggregates can be detected in these mice starting at 10 months of age [28]. The rationale behind using the young TDP-43 transgenic mice was to explore whether a subtle (2.3–3-fold) overexpression of TDP-43 represents an additional risk factor in the context of mild repetitive TBI, as well as to observe whether it would predispose and/or trigger more intense neurodegeneration, as observed in the model of stroke [44]. Furthermore, there is growing evidence that exposure to repetitive mTBI is associated with an increased risk for ALS/FTLD, especially in a subset of vulnerable individuals with genetic predisposition [12,45,46,47,48]. Furthermore, since the results of our previous study suggested that genetically acquired TDP-43 dysregulation might predispose the OT to more intense acute and subacute damage following repetitive mTBI [27], we wanted to further explore whether TDP-43 proteinopathy is associated with marked long-term posttraumatic changes in the mentioned brain structure, as well as in the investigated nuclei of the visual pathway. We used a clinically relevant model of repetitive TBI that includes the elements of acceleration/deceleration and rotational injuries of the freely moving mouse head and body resulting in diffuse brain damage. This method produced mild injuries with no skull fractures, intracranial bleeding, respiratory arrest, or seizures, and the mice quickly recovered and demonstrated normal behavior following head impacts.

### 3.1. Repetitive mTBI Induced Neurodegeneration, Axonal Injury, and Gliosis in the Optic Tract in Wild-Type and TDP-43^G348C^ Mice at 6 Months Following the Last Head Impact

In our previous research, we demonstrated that the OT was the only damaged brain structure in the injured wild-type and TDP-43 transgenic mice already on the first day following the last head impact, lasting up to the end of the first week, suggesting early vulnerability of this structure to the investigated type of injury [27]. In the current study, we histologically detected neurodegeneration using Fluoro-Jade C staining and degenerating, argyrophylic, and swollen axons using neurosilver staining in the OT of the traumatized animals of both genotypes at 6 months after repetitive mTBI, indicating chronic axonal posttraumatic damage. Our results are in agreement with some previous studies in which the destruction of this structure in rodents was reported in different models by using various injury paradigms and time points of 1, 3, 7, or 60 days, 3 or 10 weeks, and 8 or 12 months following repeated mTBI [27,31,33,42,49,50,51,52]. Taking all these results together, including the fact that repetitive mTBI increases the sensitivity of the brain to each subsequent trauma, it is plausible that the time for the recovery between individual impacts was insufficient in the experimental models and protocols used in the mentioned investigations, resulting in neurodegeneration and axonal degeneration months after the final injury. Increased sensitivity and vulnerability of the OT to the damage induced by repeated mTBI may be a consequence of its position below the brain, as well as of its anatomic characteristics. Specifically, it consists of very long myelinated axons which are susceptible to compression in the optic canal during the direct injury, as well as to tension and torsion during acceleration and deceleration forces caused by the hits [53]. Moreover, the blood supply of the optic nerve arrives from pial arteries. Its swelling, induced by repeated head traumas, may cause localized ischemic injuries that may additionally contribute to the OT damage [31]. Moreover, neurodegenerative changes in the proximal part of the visual system, including the optic nerves and the optic chiasma, and decreased cellularity in the ganglion cell layer of the retina were previously described in mice at different time points following repetitive mTBI [40,41,42,50].

The current study is the first in which the neural and axonal degeneration in the OT of TDP-43 transgenic mice was investigated at a chronic time point following repetitive mTBI. Neurodegeneration was found in the injured TDP-43^G348C^ animals compared with the related sham mice. Contrarily, significant differences in Fluoro-Jade C intensity were not observed between traumatized transgenic TDP-43 mice compared with wild-type animals, suggesting that human genetic TDP-43 background did not affect chronic damage of this structure.

In order to detect other chronic effects of repeated head impacts on the surviving axons of the OT, we investigated the levels of their myelination and the expression of the cytoskeleton NfL protein. Myelin preservation was determined by LFB and MBP stains and their quantification. Reduced LFB staining was evident in some parts of the OT in the injured mice of both genotypes. Furthermore, there was no significant difference in the staining density between the traumatized wild-type and transgenic TDP-43 mice compared with their related sham or between injured wild-type and TDP-43^G348C^ animals in this brain structure at 6 months after head traumas. Previously, no changes in LFB-positive staining were observed in the brains of mice at 6 months following the first injury in the model of repetitive mTBI induced by electromagnetic controlled impact device [32], in which local areas of reduced myelination were described in the optic nerve at 3 and 13 weeks after the last head trauma [40,41]. To our knowledge, the level of myelination was not previously investigated in the OT in the models of repeated mTBI in TDP-43 transgenic animals.

In our experiments, no changes were observed in the MBP immunoreactivity and optical density in the OT of the injured wild-type and TDP-43 transgenic mice compared with related controls or in traumatized wild-type compared with TDP-43^G348C^ animals. Taken together, the results of this study obtained by the LFB and MBP staining demonstrated that repetitive mTBI did not significantly affect myelination of the surviving axons of the retinal ganglion cells at 6 months after the final head impact and that the transgenic genotype did not influence it. In addition, Gangolli et al. [54] did not detect Myelin Black Gold staining in the OT 1 year following injury induced by CHIMERA in mice. In the same experimental model, MBP immunoreactivity was not altered in the OT 7 days after the final injury [51].

NfL chain is abundantly expressed in the long and large-caliber myelinated white-matter axons, and it is considered a promising candidate biomarker of axonal injury in different diseases of the central nervous system [55], including repetitive mTBI [56,57,58]. Increased exosomal and plasma levels of NfL chain have been detected in humans even years following repeated head traumas, suggesting chronic, long-term axonal dysregulation and degeneration induced by sustained brain injuries [57].

To our knowledge, our study is the first in which NfL chain staining was investigated in the OT at a later time point following repetitive mTBI. We did not detect statistically significant differences in the levels of this protein’s optical densities between the injured wild-type group and related sham 6 months after the final head trauma. Similar results were obtained by Cheng et al. [33] and Vonder Haar et al. [52], who also reported no differences in the Nf medium and heavy chains or the NfL chain staining in the OT between traumatized and sham mice at chronic post-injury time points in the CHIMERA model. Taking into account the results of our and other mentioned animal studies, it can be suggested that the changes in the NfL chain protein were not evident at later time points following repetitive mTBI. This could be due to previous death of the affected neurons and preserved cytoskeletal NfL architecture in surviving axons. Contrarily, axonal swellings and varicosities in the OT of the traumatized mice were observed 2 days after the injury induced by the CHIMERA method, but they disappeared by the seventh day following the last impact [59]. Taking all abovementioned results regarding the changes of the NfL following repetitive mTBI, it seems that this protein can be used as a brain marker of early axonal damage in animal models, in contrast to the human studies in which it has been detected in the blood 1 h to years after repeated head traumas [56,57,58].

Among other roles, under physiological conditions, TDP-43 binds and stabilizes NfL mRNA, regulating its transcription, metabolism, and axonal transport [60,61,62]. Contrarily, TDP-43 dysregulation, observed, e.g., in FTLD, is associated with NfL alterations and white-matter pathology [63]. In a recent study, Kumar et al. [64] found that cytoplasmic TDP-43 accumulation in mice expressing ALS-linked human TDP-43^A315T^ mutant caused marked suppression of mRNA translation for NfL, Nf medium, and α-internexin, resulting in a decrease in the levels of these proteins at 12 months of their age when they exhibited TDP-43 proteinopathy in cortical neurons. In our research, we were interested if repetitive mTBI affects NfL chain staining intensity in the OT of mice presenting with cytoplasmic TDP-43 aggregates in the spinal cord starting at approximately 10 months of age and increased pathological TDP fragment in the brain and spinal cord at 10 months of age [28]. We did not detect any significant changes in NfL chain staining in the OT of injured TDP-43^G348C^ mice compared to the related sham group or between traumatized wild-type and TDP-43 transgenic animals at 6 months following repeated head injury, suggesting that repetitive mTBI and the investigated genotype did not affect the structure and, consequently, function of this neuronal cytoskeletal protein in the used experimental conditions. Because the age of our experimental animals was approximately 8.5 months at the time of the experiments, it remains to be investigated whether there are age-dependent changes in NfL chain expression in mice of the tested TDP-43 genotype.

Neuroinflammation is one of the most important processes developing after head trauma that may have beneficial or detrimental effects in the acute TBI [65]; however, if it is chronic, it usually contributes to the brain damage [66,67,68]. In the current study, pronounced microglial and astrocytic response to the repetitive mTBI was found in the OT of the injured wild-type and TDP-43 transgenic mice related to their sham groups, suggesting chronic neuroinflammation of this structure as a result of synergistic exacerbating effects of repeated head traumas that provoked an increase in inflammatory responses during short time periods between each injury. In our previous study, using the same closed head weight drop method, significant microgliosis and astrocytosis were also demonstrated in traumatized wild-type and TDP-43^G348C^ mice, in the acute and subacute posttraumatic periods [27]. Our previous and current results regarding gliosis in the OT following repetitive mTBI are in agreement with those obtained in animal studies in which the rodents were subjected to impacts induced by other methods and in which various head trauma protocols and different posttraumatic time points from 1 to 365 days after the injury were used [31,33,43,49,50,51,52,59,69,70]. Our study found no differences in the microglial and astrocytic hyperactivities between injured transgenic TDP-43 and wild-type mice, suggesting that the investigated genetic background did not affect inflammatory parameters used in this research. Taking all the abovementioned results together, it may be suggested that the OT is particularly susceptible and vulnerable to neuroinflammation induced by repetitive mTBI.

### 3.2. Repetitive mTBI Did Not Cause Neurodegeneration, Changes in the Responses of Glial Cells, and Synaptic Reorganization in the Lateral Geniculate Nucleus and the Superior Colliculus in Wild-Type and TDP-43^G348C^ Mice at 6 Months Following the Last Head Impact

To the best of our knowledge, we are the first group to explore possible chronic damage, glial activity, and synaptic organization in the LGN and the SC following repetitive mTBI in mice of both genotypes.

The LGN, situated in the thalamus, was reported to receive external visual information mostly by the axons of the retinal ganglion cells, conducting them to the visual cortex [71]. The SC has a laminar structure, and its three superficial layers are primarily visual sensory in nature [72]. In mice, the SC also receives the projections, but from at least 70% [73] and possibly even approximately 88% of the retinal ganglion cells [74].

In the current study, no signs of neurodegeneration in the LGN and only a few scattered Fluoro-Jade C-positive signals in the superficial SC were detected in the injured mice of both genotypes. Additionally, when using cresyl-violet, no differences were revealed in the neuronal density between the injured wild-type and TDP-43 transgenic mice compared with their related sham or between traumatized wild-type and TDP-43^G348C^ mice. Considering the abovementioned results, we suggest that repetitive mTBI did not induce chronic damage of the target nuclei and that the investigated genotype did not influence it. Furthermore, reactive microgliosis or astrocytosis were not detected. Previously, microglial infiltration and activation in the SC were found 7 days after the last head trauma [50]. In the same research, the injury of the LGN was mentioned [50]. Both results were obtained using a method of repeated head traumas different from ours.

The central nervous system has the capacity of neuroplasticity and recovery following different insults that include various processes, ranging from molecular, cellular, and synaptic to global [75]. Some of these processes are associated with changes in the expression of different specific proteins, synaptogenesis markers, and synapse remodeling, such as SYP [76,77,78,79]. In our study, no differences in the SYP immunostaining intensities between the experimental groups were detected, suggesting that repetitive mTBI or the genotype did not induce synaptic perturbations in the LGN or the SC at 6 months following repetitive mTBI. To our knowledge, synaptogenesis markers have not been previously studied in the LGN and the SC following repeated head traumas.

### 3.3. Repetitive mTBI Did Not Affect Barnes Maze Task in Wild-Type and TDP-43^G348C^ Mice at 6 Months Following Repetitive mTBI

In the current research, we evaluated the effects of repetitive mTBI on the possibility of successful performance in the Barnes maze task. The Barnes maze task is usually used as a spatial learning/memory test in rodents taking advantage of their innate behavior to run away from brightly illuminated to dark areas [80]; however, in our study, it was applied primarily to test the functional status of the visual system following repeated head traumas. No differences in the time to escape to the target hole or in the time spent in the target quadrant were observed between the experimental groups, suggesting that neuronal damage, axonal damage, and neuroinflammation detected in the OT at 6 months after the last head trauma, as well as genetic TDP-43 background, did not influence the performance in the Barnes maze task. In addition, the Barnes maze task, as a cognitive test, was performed in some other studies in rodents of different ages and genotypes in which various repetitive mTBI methods, severity of the injuries, protocols, tested parameters, and posttraumatic periods were used, which is the reason why the obtained results are not consistent and hardly reciprocally comparable [33,59,69,81,82,83]. Previously, it was shown that TDP-43^G348C^ animals exhibited a significant reduction in the time spent in the target quadrant and increased primary errors in the Barnes maze test as compared with age-matched wild-type mice at 10 months of age [28]. In our study, all mice, including these transgenic traumatized and control animals, performed the task equally at approximately 8.5 months of age. Further studies are needed in order to explore age-dependent behavior of TDP-43^G348C^ mice in the Barnes maze task.

In summary, current study results suggest that repetitive mTBI induced damage of the OT, but did not affect the nuclei that transmit information from the retinal ganglion cells to the visual cortex, as well as mouse behavior that includes preserved vision, at 6 months after the last head impact. In addition, genetic TDP-43 background did not influence the assessed neuropathology and behavior in experimental animals. This study could improve our knowledge and understanding of chronic neuropathological changes in the visual system following repetitive mTBI and the role of TDP-43 proteinopathy in these processes.

## 4. Materials and Methods

### 4.1. Animals and Treatment

This study was performed on wild-type C57BL/6J and transgenic TDP-43^G348C^ male mice of C57BL/6J background. At the beginning of the experiments, the mice were 9–11 weeks old. Transgenic TDP-43^G348C^ mice were obtained from the University Laval, Quebec, Canada, and the colony was raised in the Laboratory for Mice Breeding and Engineering Rijeka, Faculty of Medicine Rijeka, University of Rijeka, Croatia. All the experimental procedures were performed according to the Faculty’s Ethical Committee approval and in accordance with the Croatian laws and rules (NN 135/06; NN 37/13; NN 125/13; NN 39/17), as well as the guidelines of the European Community Council Directive (86/609/EEC). Mice were maintained in the animal facility of the Faculty’s Department of Basic and Clinical Pharmacology and Toxicology in temperature- and humidity-controlled holding rooms, with an alternating 12 hour light/dark cycle. Fresh water and standard rodent chew were available to animals ad libitum.

Mild brain traumas were induced using the closed head weight drop method previously described by Kane et al. [84]. In brief, mice were anesthetized with 3.5% isoflurane in a nitrous oxide/oxygen (2:1) mixture in an induction chamber and rapidly positioned on aluminum foil placed over a Plexiglas box, lined with a sponge. The box was situated beneath the vertical metal tube of the apparatus. A steel weight (1.2 cm diameter, mass 97 g), set above the mouse head and between the ears, was pulled rapidly upward to 1 m height and released. Following the impact, the mice fell down through the foil and onto the surface of the sponge, all while rotating their bodies by 180° horizontally. After each mild brain trauma, the mice were returned to their holding cages to recover. In our experiments, mice rapidly recovered and showed normal interactions with other animals without demonstrating signs of pain or indisposition nor resistance to manipulations after mTBI. We did not observe respiratory arrest or seizures in any of the tested animals. For the control group, sham treated animals were only briefly anesthetized without receiving impacts. Sham procedures or mild brain traumas were repeated twice daily, in intervals of 6 h, for five consecutive days, i.e., a total of 10 impacts. Mice were euthanized at 6 months after the final impact or sham procedure.

### 4.2. Polymerase Chain Reaction

In the transgenic animals, the presence of TDP-43^G348C^ transgene was determined by polymerase chain reaction (PCR) as previously described [28]. GoTaq^®^ G2 Green Master Mix (Promega Corporation, Madison, WI, USA) and the primers CTCTTTGTGGAGAGGAC and TTATTACCCGATGGGCA (Metabion international AG, Planegg, Germany) were used for the reaction.

### 4.3. Tissue Preparation

For histochemistry purposes, the animals were anesthetized with xylazine and ketamine mixture and transcardially perfused, first with phosphate-buffered saline (PBS) and then prefixed with 4% paraformaldehyde in PBS. Their brains were dissected and post-fixed for 20–22 h in the same fixative solution at room temperature and then embedded in paraffin. Brain sections were cut to 3 μm thickness. For the analyses of the OTs, coronal sections ranging from bregma +1.18 to −2.30 [85] were used. To detect changes in the LGN, we analyzed coronal sections cut at approximately −2.46 from bregma, and the SC nuclei were investigated at around −3.52 from bregma [85].

### 4.4. Fluoro-Jade C Staining

The slides were deparaffinized in xylene, rehydrated in ethanol and water, and then treated for 10 min with a 0.06% potassium permanganate solution. Sections were rinsed twice with distilled water (dH_2_O) for 1 min and incubated in 0.0001% Fluoro-Jade C (Chemicon, Millipore, Billerica, MA, USA) staining solution for 20 min in the dark. After that, they were washed in dH_2_O thrice per minute and dried on a hot plate on 50 °C for 20 min. Sections were dehydrated in xylene two times for 10 min, mounted in *Entellan^®^* (Merck Millipore, Billerica, MA, USA), and coverslipped. Stained sections were examined by epifluorescence microscopy using the appropriate light filter cube (Olympus BX 51 microscope with Olympus DP 70 digital camera, Olympus, Tokyo, Japan).

Quantification of Fluoro-Jade C intensity in the OT was done on microphotographs taken at ×400 final magnification; for each animal, two images were used for the analyses. Within each microphotograph, three ROIs of 0.0057 mm^2^ were analyzed. By subtracting the background fluorescent intensity from those ROIs, we could determine only degenerating axons within that field.

### 4.5. Bielschowsky Silver Staining

Bielschowsky silver staining is a method that can be used to detect nerve fibers and stain axons, neurofibrils, and senile plaques in the central nervous system. Brain sections were deparaffinized, dehydrated, and then immersed for 15 min in the solution with 20% silver nitrate, preheated at 37 °C. Sections were then washed in distilled water, after which they were submerged in silver ammonia solution for 15 min, all at 37 °C. Next, slides were developed by placing them in 50 mL of distilled water with eight drops of both ammonium hydroxide and developer stock (8% *v*/*v* formaldehyde, 0.5% *w*/*v* citric acid, 0.1% *v*/*v* nitric acid) for 2 min maximum, i.e., until their color changed to the desired intensity of brown. Following the washing step in distilled water, sections were immersed in 5% sodium thiosulfate for 5 min at room temperature, washed with tap water for 5 min, dehydrated, cleared, and mounted with Entellan*^®^*.

Microphotographs of the OTs stained with Bielschowsky silver stain were taken at ×400 magnification using an Olympus BX 51 microscope with an Olympus DP 70 digital camera (Olympus, Tokyo, Japan).

### 4.6. Luxol Fast Blue Staining

LFB staining of the OT was performed to determine the degree of myelination. Following deparaffinization and rehydration, brain sections were stained with LFB solution at 56 °C overnight. The next day, slides were rinsed with 95% ethanol and distilled water, after which they were differentiated in the lithium carbonate solution for 10–15 s, and then immersed briefly in the 70% ethanol three times. Following that, slides were washed in distilled water, dehydrated through rising ethanol concentrations, cleared in xylene, and mounted with Entellan*^®^*.

LFB-stained sections were photographed at 200× magnification using an Olympus BX 51 microscope with an Olympus DP 70 digital camera (Olympus, Japan).

Myelin densities on LFB stained photographs were quantified by using the ImageJ software (NIH, Bethesda, Md, USA), according to the protocol described by Underhill et al. [86] with modifications suggested by Khodanovich et al. [87]. Briefly, mean intensities of the red channel in a region of interest (ROI), i.e., in the OTs, were measured from RGB images as a quantity characterizing the complementary blue channel saturation. The background mean intensity of the red channel was also measured on each photograph in ROIs outside the brain tissue, which served for the calculation of the background correction factor. LFB optical density (in %) was calculated for each ROI according to the following formula: LFB density = 100 × (1 − (red channel intensity/background intensity)).

### 4.7. Immunofluorescence/Immunohistochemistry

To investigate the expression of the proteins of interest, we used immunofluorescent labeling in combination with DAPI nuclear counterstaining or immunohistological staining visualized with 3,3′-diaminobenzidine (DAB) chromogen (Dako).

After deparaffinization and rehydration of slides, antigen retrieval was achieved by heat-induced epitope retrieval procedure in the citric acid buffer (10 mM, pH 6.0). Nonspecific binding sites were blocked with Tris-buffered saline (TBS) containing 5% bovine serum albumin (BSA) and 0.025% Triton X-100. Slides were incubated overnight at 4 °C with primary antibodies, as listed in Table 1.

For the sections immunolabeled and visualized by using the DAB chromogen, the brain slices were incubated with an appropriate biotinylated secondary antibody (Table 1), diluted in the antibody solution buffer for 1 h at RT, followed by the streptavidin–HRP conjugate for 30 min at RT. Following the application of DAB, reaction with HRP produced a brown precipitate. The slides were then dehydrated, immersed in xylene, and mounted. For the immunofluorescence labeling, appropriate fluorochrome-conjugated secondary antibodies were applied for 1 h at RT. Cell nuclei were counterstained with DAPI, and the slides were mounted in anti-fade mounting medium.

Immunolabeled sections were examined by light or epifluorescence microscopy (Olympus BX 51 microscope with Olympus DP 70 digital camera, Olympus, Tokyo, Japan).

Quantification of neurodegeneration and the glial response in the OTs was done on Fluoro-Jade C-stained and Iba1- or GFAP-immunolabeled coronal sections cut in the range of −1.34 to−2.30 from bregma [85] using ImageJ software (NIH, Bethesda, Md, USA). Quantification of microgliosis and astrocytosis was made by measuring the percentage (%) of the Iba1- or GFAP-immunoreactive areas. Microphotographs of two sections from each animal, at ×400 magnification, were transformed to 8 bit images and auto-thresholded (0 being white and 255 being black), which enabled differentiating positive immunoreactions from the background and calculating the immunoreactive area fraction. A region of interest (ROI) was drawn around the OT. The area fractions were averaged for each animal and each experimental group.

In the OTs, quantification of the DAB staining intensity was also done with ImageJ software. Briefly, mean gray values were collected from the ROIs selected in the images of the OTs, and optical density (OD) was calculated with the following formula: OD = log(max gray intensity/mean gray intensity).

In the LGN and the SC, we evaluated the intensity of SYP immunofluorescent staining. Conditions of the microscopy and photography were maintained constant throughout the experiment, and immunoreactivity was quantified by measuring the integrated optical density. In the mentioned nuclei, we also evaluated the microglial response by counting the number of Iba1-stained cells in the immunofluorescently labeled sections.

### 4.8. Cresyl-Violet

Cresyl-violet (Nissl) staining was used to detect the effects of repetitive mTBI on the number of neurons in the investigated nuclei of the visual system. Deparaffinized and rehydrated slides were stained with 0.1% cresyl-violet acetate (Sigma Aldrich, St Louis, MO, USA) by incubation for 10 min at room temperature. Differentiation of the brain tissue sections was done by immersing the slides in 95% alcohol with glacial acetic acid. Finally, brain sections were dehydrated in alcohol, cleared in xylene, and mounted with Entellan*^®^*.

Microphotographs of the cresyl-violet stained sections were taken at approximately −2.46 from bregma for the LGN and at −3.52 from bregma for the SC [85], at ×400 magnification, using an Olympus BX 51 microscope equipped with an Olympus DP 70 digital camera (Olympus, Tokyo, Japan). With the help of the ImageJ software, neuronal density estimation in the investigated nuclei was carried out by a blind investigator using the random simple counting method. Two to four random ROIs were taken from at least two serial cuts of the selected areas and used to estimate the number of the neurons. Only cells with visible nuclei were counted.

### 4.9. Barnes Maze Task

To test the behavior of experimental animals following repetitive mTBI that requires preserved vision, the Barnes maze task was performed. A homemade maze was situated in an experimental room with distinct visual cues. The apparatus consisted of an elevated circular platform 92 cm in diameter with 20 escape holes, 5 cm in diameter, spaced evenly around the perimeter, and an escape box placed underneath target hole. The maze was divided into quadrants consisting of five holes each, and the target hole was located in the center of the target quadrant. Visual cues enabled mice proper space orientation to learn and reach the target hole and quadrant. On the habituation day, on posttraumatic day 167, the mouse was placed under the black start chamber in the center of the platform for 10 s. After that, the box was removed and the animal was trained to find and enter the escape box. The mouse was allowed for 1 min inside the escape box and then returned to the holding cage. After this procedure, the animal was subjected to four daily described training trials that lasted 3 min each, with an intertrial interval of 15 min, on days 167 to 170 after the last mTBI. The test, during which escape box was removed from the maze, was conducted 24 h after the last training day and lasted 1.5 min per mouse. Seven days after the test, mice were retested. Using video tracking software (ANY-maze, Stoelting Europe, Dublin, Ireland), we recorded the time taken by the individual mouse to reach the target hole, as well as the time that each mouse spent in the target quadrant during the test and retest.

### 4.10. Laboratory Data and Statistical Analyses

Data were collected using the Microsoft Excel 2016 (Microsoft Corp., Redmond, WA, USA) and, when necessary, corrected for between-session variation as described previously [88]. All the statistical analyses were performed in the Statistica software version 13.5 (StatSoft Inc., Tulsa, OK, USA). According to the normality of the results, we used the nonparametric Kruskal–Wallis test followed by Mann–Whitney U test for all analyses, except the analyses of the time spent in the target quadrant for the Barnes maze task performance, for which parametric one-way analysis of variance test was utilized. Results are expressed as means ± standard error of the mean. In all comparisons, *p* < 0.05 was considered to indicate statistical significance.

## Figures and Tables

**Table 1:** List of antibodies used for the immunofluorescent (IF) and immunohistological (IHC) analyses.

**Primary Antibody**	**Dilution**	**Manufacturer** **(Reference Number)**
Rabbit anti-Iba1	1:1000 (IF)	Wako Chemicals, Richmond, VA, USA (019-19741)
Mouse anti-GFAP	1:200 (IF)	Cell Signaling Technology, Beverly, MA, USA (#3670)
Rabbit anti-MBP	1:2500 (IHC)	Abcam, Cambridge, UK (ab218011)
Rabbit anti-NfL	1:100 (IHC)	Cell Signaling Technology, Beverly, MA, USA (#2837)
Mouse anti-SYP	1:200 (IF)	Santa Cruz Biotechnology, Santa Cruz, CA, USA (sc-17750)
**Secondary Antibody**	**Dilution**	**Manufacturer** **(Reference Number)**
Goat anti-rabbit Alexa Fluor 594	1:200 (IF)	Abcam, Cambridge, UK (ab6901)
Rabbit anti-mouse Alexa Fluor 594	1:200 (IF)	Cell Signaling Technology (#4408)
Biotinylated goat anti-rabbit	1:200 (IHC)	Invitrogen, Carlsbad, CA, USA (65-6140)

Abbreviations: Iba1, ionized calcium-binding adapter molecule 1; GFAP, glial fibrillary acidic protein; MBP, myelin basic protein; NfL, neurofilament light chain; SYP, synaptophysin.

## Data Availability

The data that support the findings of this study are available within the article and supplemental data or upon request from the corresponding author.

## Supplementary Materials

The following are available online at https://www.mdpi.com/article/10.3390/ijms22126584/s1: Figure S1. Representative microphotographs of the optic tracts in wild-type (WT) and TDP-43^G348C^ mice at 6 months after repetitive mild traumatic brain injury (rmTBI) or sham procedure (Sham), immunostained with anti-ionized calcium-binding adaptor molecule 1 (Iba1), showing different morphological forms of microglial cells; Figure S2. Fluoro-Jade C- and cresyl-violet-stained sections of the lateral geniculate nucleus (LGN) and the superior colliculus (SC) in wild-type (WT) and TDP-43^G348C^ mice at 6 months after repetitive mild traumatic brain injury (rmTBI); Figure S3. The activity of the glial cells in the lateral geniculate nucleus (LGN) and the superior colliculus (SC) in wild-type (WT) and TDP-43^G348C^ mice at 6 months after repetitive mild traumatic brain injury (rmTBI); Figure S4. Synaptic plasticity in the lateral geniculate nucleus (LGN) and the superior colliculus (SC) in wild-type (WT) and TDP-43^G348C^ mice at 6 months after repetitive mild traumatic brain injury (rmTBI).
