# Supplementary material for: Long-Term Effects of Repetitive Mild Traumatic Injury on the Visual System in Wild-Type and TDP-43 Transgenic Mice
Source: Int J Mol Sci. 2021 Jun 19;22(12):6584. doi: 10.3390/ijms22126584 (PMC8235442; doi:10.3390/ijms22126584)
Supplement: Supplementary file 1 [file ijms-22-06584-s001.zip › Supplementary_Figures_Legends.pdf]

Supplementary Figure S1. Representative microphotographs of the optic tracts in wild-type (WT) and TDP-43^{G348C} mice at six months after repetitive mild traumatic brain injury (rmTBI) or sham procedure (Sham), immunostained with anti-ionized calcium-binding adaptor molecule 1 (Iba1), showing different morphological forms of microglial cells. Arrowheads point to Iba1-positive cells with “resting” morphology, and arrows point to Iba1-positive cells with various activated forms. Scale bar: 25 μm.

Supplementary Figure S2. Fluoro-Jade C and cresyl-violet stained sections of the lateral geniculate nucleus (LGN) and the superior colliculus (SC) in wild-type (WT) and TDP-43^{G348C} mice at six months after repetitive mild traumatic brain injury (rmTBI). Representative microphotographs of the Fluoro-Jade C stained sections of (**A**) the LGN and (**D**) the SC. (**A**) Denoted lines indicate the LGN. (**D**) Arrows point to Fluoro-Jade C positive staining in the SC. Scale bar for (**A**) the LGN: 200 μm, and (**D**) the SC: 50 μm. (**E**) The histogram shows the intensity levels of the fluorescent staining (A.U./μm^2) in the SC of WT and TDP-43^{G348C} mice of the control groups (Sham) and animals with rmTBI. Results are expressed as means ± SEM (N = 4). Representative microphotographs of the cresyl-violet stained sections of (**B**) the LGN and (**F**) the SC. Scale bar: 100 μm. Histograms show the number of cresyl-violet positive neurons/mm^2 in (**C**) the LGN and (**G**) the SC of WT and TDP-43^{G348C} mice of the control groups (Sham) and animals with rmTBI. Results are expressed as means ± SEM (N = 3–5).

Supplementary Figure S3. The activity of the glial cells in the lateral geniculate nucleus (LGN) and the superior colliculus (SC) in wild-type (WT) and TDP-43^{G348C} mice at six months after repetitive mild traumatic brain injury (rmTBI). Representative microphotographs of the sections of (**A**) the LGN and (**D**) the SC immunostained with anti-ionized calcium-binding adaptor molecule 1 (Iba1). Arowheads point to the “resting” microglia. Scale bar: 100 μm. Histograms show the numbers of Iba1-positive cells/mm^2 in (**B)** the LGN and (**E**) the SC of WT and TDP-43^{G348C} mice with rmTBI and related control groups (Sham). Results are expressed as means ± SEM (N = 3–5). Representative microphotographs of the sections (**C**) of the LGN and (**F**) the SC immunostained with anti-glial fibrillary acidic protein. Scale bar: 100 μm.

Supplementary Figure S4. Synaptic plasticity in the lateral geniculate nucleus (LGN) and the superior colliculus (SC) in wild-type (WT) and TDP-43^{G348C} mice at six months after repetitive

mild traumatic brain injury (rmTBI). Representative microphotographs of the sections of (**A**) the LGN and (**C**) the SC immunostained with anti-synaptophysin (SYP). Scale bar: 100 μm. Histograms show the SYP intensity (A.U.) in (**B**) the LGN and (**D**) the SC of WT and TDP-43^{G348C} mice with rmTBI and related control groups (Sham). Results are expressed as means ± SEM (N = 3–4).
