# Supplementary figures and images for: Long-Term Effects of Repetitive Mild Traumatic Injury on the Visual System in Wild-Type and TDP-43 Transgenic Mice
Source: Int J Mol Sci. 2021 Jun 19;22(12):6584. doi: 10.3390/ijms22126584 (PMC8235442; doi:10.3390/ijms22126584)

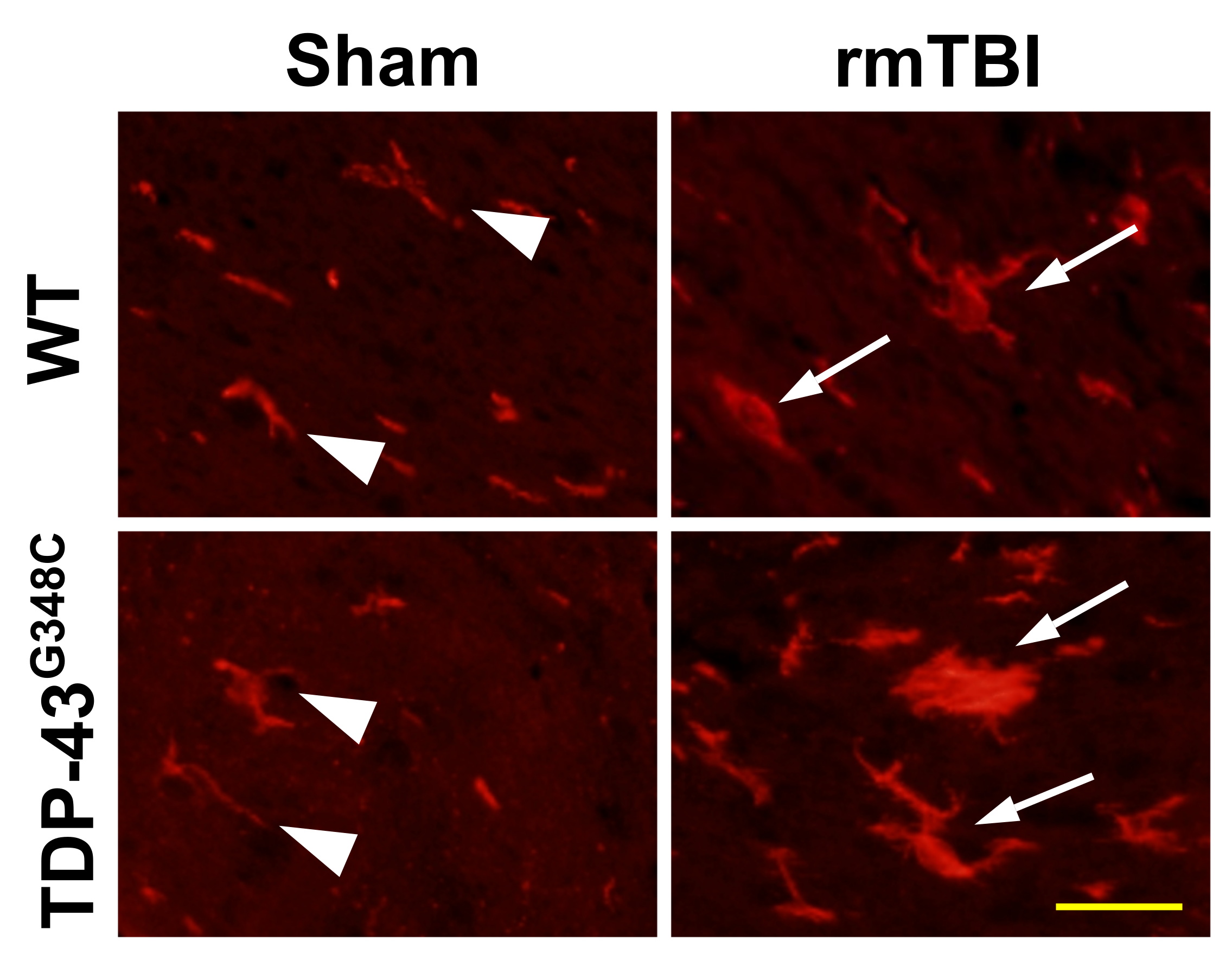

Supplement: Supplementary file 1 [file ijms-22-06584-s001.zip › Supp.fig.1.tif]

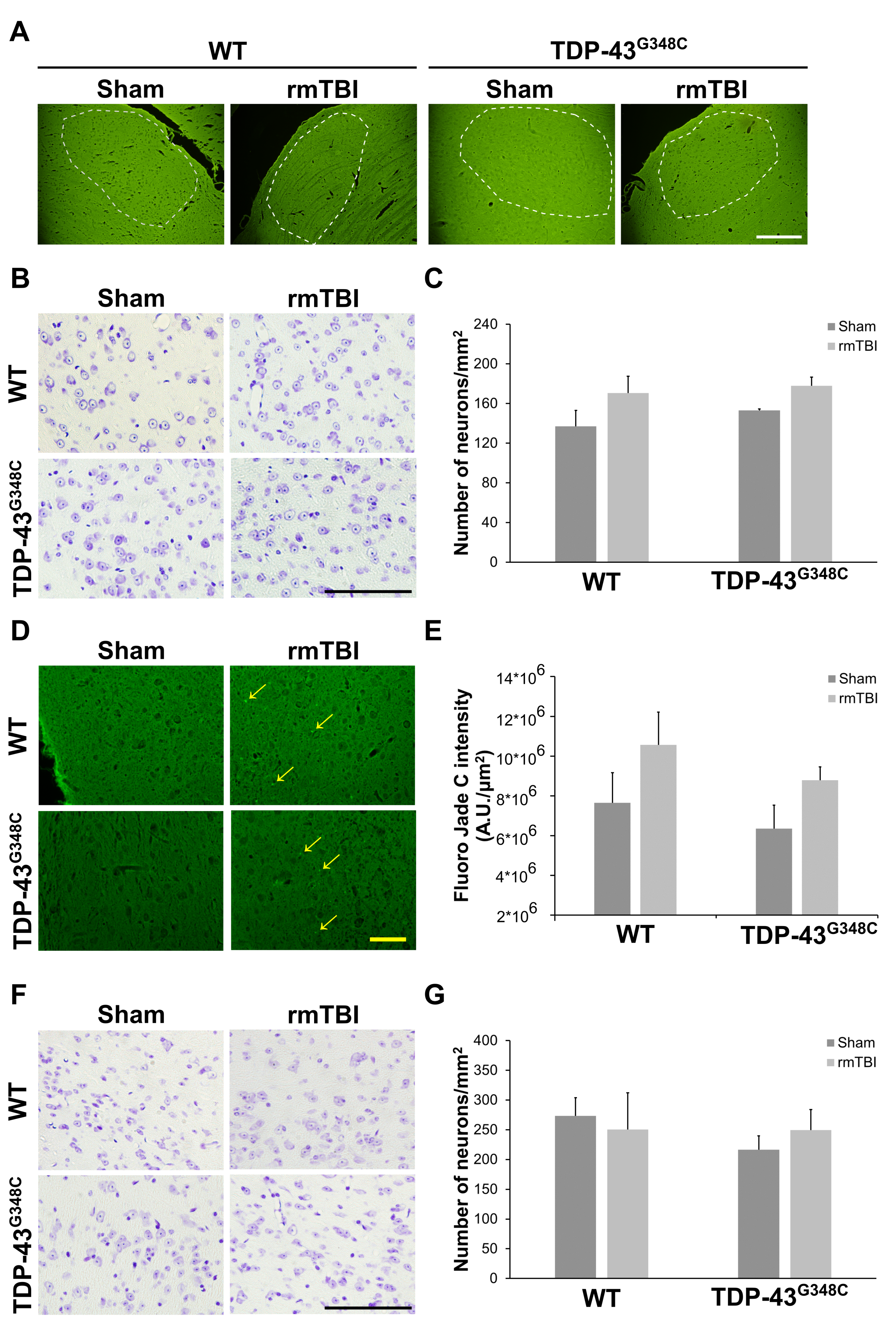

Supplement: Supplementary file 1 [file ijms-22-06584-s001.zip › Supp.fig.2.tif]

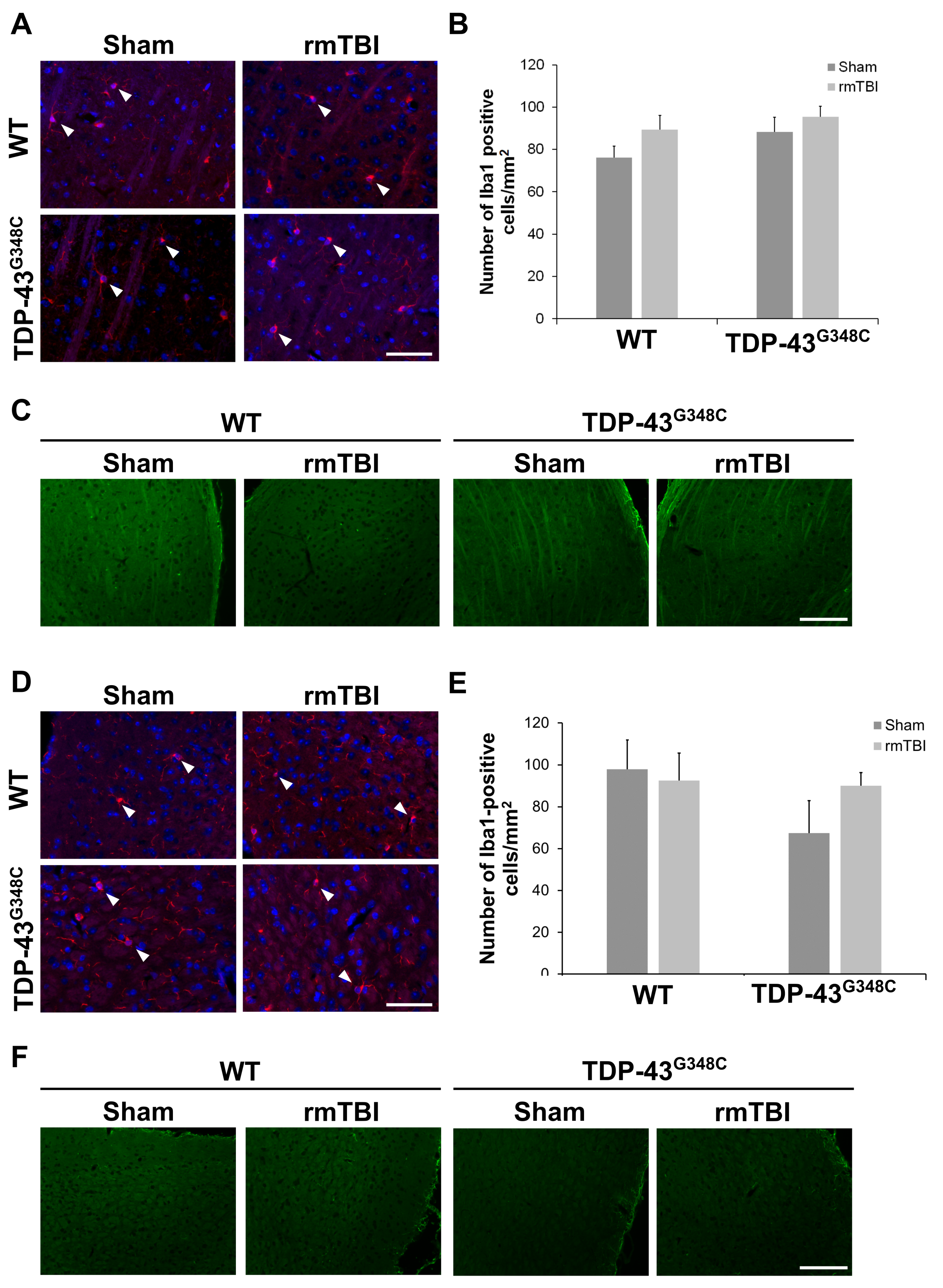

Supplement: Supplementary file 1 [file ijms-22-06584-s001.zip › Supp.fig.3.tif]

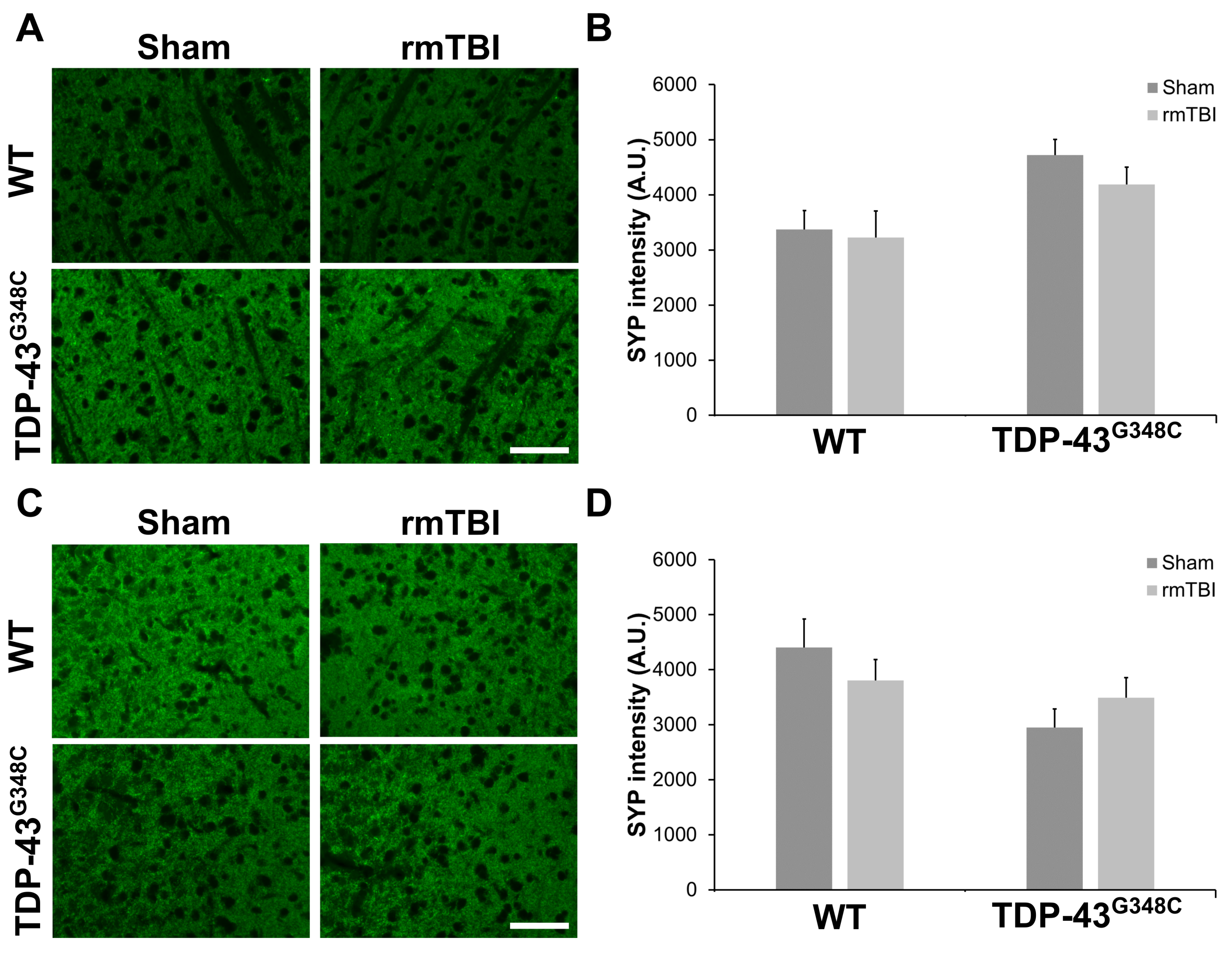

Supplement: Supplementary file 1 [file ijms-22-06584-s001.zip › Supp.fig.4.tif]
